# The clinical and demographic profile of women living with HIV admitted to the acute unit at Stikland Psychiatric Hospital

**DOI:** 10.4102/sajhivmed.v22i1.1159

**Published:** 2021-03-16

**Authors:** Jean-Marie le Roux, Lina Groenewald, Karis Moxley, Liezl Koen

**Affiliations:** 1 Department of Psychiatry, Faculty of Medicine and Health Sciences, Stellenbosch University, Cape Town, South Africa

**Keywords:** ART adherence, HIV, female, neurological disorders, psychiatry, South Africa

## Abstract

**Background:**

There is a paucity of research on the clinical profile of women living with human immunodeficiency virus (HIV) (WLWH) admitted with acute mental health illness. Existing studies are small and did not look at factors that could have an impact on medication adherence. As a first step to inform service delivery for this vulnerable population, a thorough understanding of the composition and needs of these patients should be identified.

**Objectives:**

To describe the socio-demographic and clinical profile that could have an influence on the antiretroviral therapy (ART) adherence of WLWH at an inpatient psychiatric unit.

**Methods:**

In this retrospective audit, the medical records of all WLWH (18–59 years of age), discharged from the acute unit at Stikland Psychiatric Hospital, were reviewed over a 12-month period.

**Results:**

Of the 347 female patients discharged, 55 patients were positive for HIV (15.9%). The majority of them were unmarried (78.2%), unemployed (92.7%), had a secondary level of education (Grade 8–10) (58.2%), lived with a family member (83.6%) and had children (61.8%). The most common psychiatric diagnosis on discharge was substance use disorder with 78.2% of patients being categorised as substance users. Interpersonal violence was only reported by 5.5% of patients. Although most patients performed poorly on the Montreal Cognitive Assessment (MoCA) and International HIV Dementia Scale (IHDS), only 12% of patients received a diagnosis of HIV-associated neurocognitive disorder (HAND) upon discharge. Antiretroviral therapy (ART) was initiated in 21.8% of patients. Only eight patients had a viral load of < 200 copies/mL, indicating viral suppression.

**Conclusion:**

Our findings may inform service planning and emphasise the need for targeted intervention strategies to improve treatment outcomes in this vulnerable group.

## Introduction

The HIV Impact Assessment Summary Report, released in July 2018, indicated that approximately 7.9 million people in South Africa were living with human immunodeficiency virus (HIV) in 2017.^1^ Women seem to be a particularly vulnerable population as far more females (26.3%) have HIV compared to males (14.8%),^1^ likely because of innate biological susceptibility and social factors.^2^ There is a high burden of mental illness in South Africa, especially in the Western Cape Province, which has the highest lifetime prevalence rate of mental disorders in the country.^3^ This high burden is in part attributable to the comorbidity that exists between mental, neurological and substance disorders, and other chronic medical conditions, such as HIV.^4^

The prevalence of HIV in psychiatric patients is higher than in the general population, likely because of the complex relationship between HIV and mental illness.^5,6,7^ Firstly, having a mental illness is a known risk factor for contracting HIV, with research showing that a significant number of adults with severe mental illness engage in illicit substance use and high-risk sexual behaviours.^8,9^ Secondly, comorbid psychiatric conditions are associated with immunologic changes that result in more rapid progression to AIDS and death.^10^ Conversely, having HIV may drive the development of comorbid psychiatric conditions because of lived experiences of the stigma and the HIV-associated opportunistic infections and their treatment.^11^ Antiretroviral therapy (ART) is known to have neuropsychiatric side effects,^12^ and the HIV itself has a direct effect on the brain.^13,14,15^

Antiretroviral therapy is known to have numerous benefits when adherence is optimal as is evident from the considerable decrease in HIV-related deaths and co-morbidities since the development thereof in 1996.^16^ The Southern African ART guidelines, released in 2017, advise that all individuals diagnosed with HIV must be started on treatment as soon as possible regardless of CD4 count.^17^ These guidelines have had a substantial impact on the management of people living with HIV (PLWH) in the acute psychiatric setting, as patients are often first diagnosed with HIV when admitted to a psychiatric ward.

Sustained virological suppression through ART is essential for reducing HIV-related morbidity and mortality, making non-adherence a critical issue to address.^18^ A meta-analysis of several studies that have assessed ART adherence in sub-Saharan Africa showed that PLWH achieved relatively high levels of adherence.^19^ However, these findings might not reflect the true prevalence of poor-adherence, as some studies included in the meta-analysis made use of potentially unreliable methods to measure adherence, including patient self-report or pharmacy claims. General treatment adherence amongst psychiatric patients has been shown to be problematic,^20,21^ so we might expect that their adherence to ART is also poor. Several studies have shown an increased risk of ART non-adherence in PLWH with comorbid psychiatric disorders like depression, anxiety and substance use disorders.^18,22^ Therefore, PLWH with comorbid psychiatric disorders may be at an even greater risk of defaulting on their treatment.

This is of grave concern, especially in the light of newly diagnosed PLWH being initiated on ART whilst inpatients and then discharged with no HIV-specific interventions in place to assist with adherence to ART. Intervention strategies to improve adherence to ART amongst PLWH have been shown to be effective^23^ with cognitive behavioural therapy (CBT) in these patients who have depression as one such example.^24,25^ However, to develop targeted intervention strategies in any context, especially in a severe mental illness cohort, an in-depth understanding of the clinical, medication-related, social and patient-related risk factors that could contribute to non-adherence is required.

There is a paucity of research on the clinical profile of women living with HIV (WLWH) and severe mental illness, especially in the South African setting. Existing studies are small and did not look at factors that could have an impact on adherence.^7,26,27^ Given biological susceptibility and high prevalence of HIV amongst women, there is a critical need to assess factors that may contribute to non-adherence amongst women, and the aim of this study was to take a first step towards addressing this issue. Focusing primarily on demographic and clinical characteristics that could influence adherence to ART, we describe a cohort of WLWH and admitted to an acute psychiatric unit over a 12-month period. Going forward, these data could provide a starting point for the development of strategies to improve ART adherence and inform service delivery for this vulnerable group.

## Methods

### Study design

This study involved a retrospective analysis of the records of patients with a comorbid diagnosis (new or previously known) of HIV discharged from the acute female psychiatric unit at Stikland Hospital over the 12-month period, 01 June 2018 to 31 May 2019.

### Study setting

Stikland Psychiatric Hospital, located in Bellville, Western Cape, provides services to a catchment area that represents approximately one-third of the province’s population. The hospital offers 423 beds, and the acute female inpatient unit comprises two wards with a total of 59 state-funded beds. In addition to receiving full psychiatric and medical assessment, inpatients whose HIV status is unknown are offered testing, including standard consent and counselling procedures. A urine toxicology test is also done on most patients to screen for the use of illicit substances.

As per the current ART guidelines, the CD4 count of patients is assessed at diagnosis and then at 12 months after ART initiation. For those patients with CD4 counts < 200 cells/mm^3^, CD4 is assessed annually until it reaches > 200 cells/mm^3^. Patients who are newly diagnosed with HIV or who were diagnosed within the past year would have had only one CD4 count available in their charts, which was then seen as their most recent CD4 count as well as their nadir CD4 count (lowest CD4 count a patient ever had).

Viral load monitoring is done at month 4, 12 and then annually after ART initiation for patients on first-line treatment and at month 6, 12 and then annually for patients on second- and third-line treatment.^28^

If possible, a Montreal Cognitive Assessment (MoCA) and International HIV Dementia Scale (IHDS) are done to screen for HIV-associated neurocognitive disorder (HAND) prior to discharge when patients are apsychotic and euthymic. During the reporting period, the cognitive tests were done in the patient’s first language. A diagnosis of HAND is made based on the history from the patient, the clinical findings, and the results of cognitive testing.

### Study sample

The study sample included all WLWH aged 18–59 years, who were inpatients of a psychiatric unit, and had been discharged from the acute wards at Stikland Hospital between 01 June 2018 and 31 May 2019. From a total of 347 female psychiatric inpatients, 55 patients were found to have a co-morbid HIV diagnosis and thus comprised our final sample.

### Data collection

#### Procedure

Patient files were retrieved from Stikland Hospital archives and were reviewed to identify all PLWH. Thereafter, patient’s data were extracted from the files and entered into a Microsoft Excel spreadsheet by the principal investigator. Patient anonymity was protected by using delinked number identifiers. The data extracted included demographic and psychiatric or medical clinical variables including CD4 count, viral load, results of cognitive testing (i.e. IHDS total score and MoCA total score), substance use, psychiatric diagnoses, comorbid infections, other medical comorbidities and medication (with a specific focus on ART). We accessed electronic pharmacy data to determine the number of tablets patients with HIV received upon discharge and the amount of times they had to take these daily. Treatment support was said to be present if a note was made in the file that the family was contacted by either a social worker or a doctor to discuss the importance of adherence. Substance use disorders and psychiatric diagnoses were made according to the DSM 5 criteria. Information about the disclosure of HIV status was obtained if a note in the file stated that a patient disclosed their status to a friend and/or family member. Interpersonal violence was said to be present if the patient confirmed that they were currently in an abusive relationship.

#### Measures

The MoCA was originally developed in Canada as a screening tool for mild cognitive impairment in older adults. The MoCA is scored out of 30, takes 10 – 15 min to complete and consists of 13 tasks that assess participants across six broad domains of ability and neurocognitive function.^29^ One point is added to the total score if a person has 12 years or less of formal education. Using a cut-off score of 26 or less has been found to have 90% sensitivity and 87% specificity in detecting mild cognitive impairment.^29^ Up till now, it has not been validated in South Africa.

The IHDS is a screening instrument developed specifically for HAND. It consists of three items, is scored out of 12 and takes approximately 10 min to complete. It has been validated in South Africa and showed a sensitivity of 45% and specificity of 79% at a cut-off score of 10 or less.^30^ A cut-off score of 11 or less has 53% sensitive and 80% specificity. The IHDS is not influenced by the level of education or language.^31^

### Data analysis

For descriptive analyses, nominal data were summarised as counts and frequencies, whilst numerical data were summarised as means with standard deviation. We used independent *t*-tests to examine differences in CD4 count and viral load based on MoCA and IHDS scores, which were normally distributed. Statistical significance was set at *p* < 0.05 and all analyses were performed using SPSS, version 25.

### Ethical considerations

Ethical approval was obtained from the Health Research Ethics Committee of Stellenbosch University (reference S19/06/109), and we were granted a waiver of informed consent. All data were anonymised to ensure privacy and confidentiality of participants’ personal information, assigning each participant a unique identifier.

## Results

### Socio-demographic characteristics

Of the 347 female patients discharged from the acute unit at Stikland Psychiatric Hospital between 01 June 2018 and 31 May 2019, only 55 patients were positive for HIV (15.9%). The socio-demographic characteristics of these patients are summarised in Table 1.

**TABLE 1:** Socio-demographic characteristics of women (*n* = 55).

Variable	*n*	%
**Accommodation**		
Family	46	83.6
Own	2	3.6
Other	7	12.8
**Marital status**		
Not in a relationship†	43	78.2
In a relationship‡	12	21.8
**Children**		
No	20	36.4
Yes	34	61.8
Unknown	1	1.8
**Highest level of education**		
Primary Grade 1 to 7	13	23.6
Secondary Grade 8 to 10	32	58.2
Secondary Grade 11 to 12	6	10.9
Tertiary	4	7.3
**Employment**		
No	51	92.7
Yes	4	7.3
**Disability grant**		
No	32	58.2
Yes	23	41.8
**Interpersonal violence**		
No	44	80.0
Yes	3	5.5
Unknown	8	14.5

† , Single, divorced, widowed, or separated.

‡ , Married or has a partner.

The mean age of the cohort was 30.7 years (range 19 to 46 years; standard deviation [SD] 7.4). The average length of stay in days during the current admission was 65.6 days (range 5 to 225; SD 49.7) and the mean number of previous psychiatric admissions was 2.29 (range 0 to 9; SD 2.2).

Most patients disclosed their HIV status (85%), either to a family member (*n* = 46) or to a partner (*n* = 1). Treatment support was arranged in 83.6% of cases upon discharge. Most patients (78.2%) were discharged into the care of their family members, and 16.4% of patients were discharged to a psychosocial rehabilitation facility. Interpersonal violence was indicated in 5.5% of patient folders. Upon discharge, 41.8% of patients were receiving a monthly disability grant.

#### Clinical characteristics

The average CD4 count of 54 patients who had this tested was 620.9 cells/mm^3^ (range 42 to 1252; SD 296.6). The average nadir CD4 count of 54 patients who had the test done was 492.13 cells/mm^3^ (range 40 to 1036). One patient’s CD4 count was not requested, most likely because of unintended oversight. The viral load of only 25 of the 55 patients was tested. The most recent average viral load for these patients was 30 to 703.8 copies/mL (range 20 to 367 164; SD 82442.669). Eight patients had a viral load of < 200 copies/mL, which indicated viral suppression at the time of study.^32^ Only four of the patients had a viral load of < 50, which indicates viral suppression based on the most recent guidelines.^33^ All eight patients who were virally suppressed were on first-line therapy. Of the 17 patients who were not virally suppressed, 9 were on FDC (a fixed-dose combination tablet containing tenofovir [TDF] + emtricitabine [FTC] + efavirenz [EFV]); 4 were on first-line treatment, but not FDC; and 3 were on second-line treatment. One patient refused treatment.

The mean Body Mass Index (BMI) of the 55 PLWH was 28.0 (SD 8.3). Only 9 patients (16.4%) had other medical comorbidities (not including opportunistic infections). Of those patients, metabolic comorbidities were the most prevalent (*n* = 3), followed by endocrine, neurological and pulmonary comorbidities (*n* = 2 for all). Previous opportunistic infections occurred in 23 patients (41.8% of the total sample) (Table 2).

**TABLE 2:** Frequencies of current or previous opportunistic infections in women living with HIV (*n* = 55).

Infection	*n*	%†
Pulmonary tuberculosis	7	12.7
TB meningitis	1	1.8
Primary Syphilis	11	20.0
Neurosyphilis	4	7.3
Epstein-Barr virus meningitis	2	3.6

† , Percentages, calculated to the total sample size (*n* = 55).

#### Cognitive assessment

The MoCA was done for 37 patients and the mean score was 18.1 (SD 5.6). A total of 32 patients scored lower than 26 on the MoCA, and 4 of these patients were diagnosed with HAND. A total of 15 patients had both MoCA scores and viral load data in their charts. Within this group, the viral load in patients with MoCA scores < 26 was not significantly higher than that in patients with normal cognition (Table 3).

**TABLE 3:** Comparison of viral load in patients who had both viral load and Montreal Cognitive Assessment (*n* = 15) or International HIV Dementia Scale scores (*n* = 15) in their files.

Test scores	*n*	Mean score (SD)	Viral load, copies/mL (SD)	*p*
**MoCA**				
< 26	13	16.5	49 140.9	0.849
≥ 26	2	27.5	33 262.0	
**IHDS**				
≤ 10	10	9.0	27 142.1	< 0.001*
> 10	5	11.6	86 777.2	

* , Indicates statistical significance (independent *t*-test) at *p* < 0.05.

MoCA, Montreal Cognitive Assessment; IHDS, International HIV Dementia Scale.

The IHDS was done for 34 patients and the mean score was 9.6 (SD 1.6). A total of 22 patients had IHDS scores ≤ 10, and 6 patients were diagnosed with HAND. A total of 15 patients had both IHDS scores and viral load data in their charts. The viral load in patients with IHDS scores ≤ 10 was significantly lower (*p* = 0.001) than that in patients who scored above 10 (Table 3).

A total of 36 patients had both CD4 and MoCA screening. The average CD4 count was higher in the group that scored below 26 on the MoCA, compared to the group that scored above 26 (Table 4) but this difference was not statistically significant. A total of 34 patients had both CD4 count and IHDS screening. The CD4 count was not significantly higher in the group that scored ≤ 10, compared to the group that scored > 10.

**TABLE 4:** Comparison of most recent CD4 count in patients who had both CD4 and Montreal Cognitive Assessment (*n* = 36) or International HIV Dementia Scale scores (*n* = 34).

Test scores	*n*	CD 4 count, minimum (cells/mm^3^)	CD4 count, maximum (cells/mm^3^)	CD4 count, mean ± SD (cells/mm^3^)	*p*
**MoCA**					0.628
< 26	31	148	1224	631.32 ± 308.586	
≥ 26	5	293	609	453 ± 119.097	
**IHDS**					0.341
≤ 10	22	153	1224	676.82 ± 262.849	
> 10	12	180	930	440 ± 219.393	

MoCA, Montreal Cognitive Assessment; IHDS, International HIV Dementia Scale.

The nadir CD4 count and MoCA score were available for 36 patients. The mean nadir CD4 did not significantly differ between the groups that scored ≥ 26 and < 26 on the MoCA. Nor did the nadir CD4 differ between the groups that scored > 10 and ≤ 10 (Table 5).

**TABLE 5:** Comparison of nadir CD4 in patients who had both nadir CD4 and Montreal Cognitive Assessment (*n* = 36) or International HIV Dementia scale scores (*n* = 34).

Test scores	*n*	CD4 count, mean ± SD (cells/mm^3^)
**MoCA**		
< 26	31	500.06 ± 268.206
≥ 26	5	439.8 ± 121.619
**IHDS**		
≤ 10	22	502.27 ± 224.306
> 10	12	452.92 ± 212.594

MoCA, Montreal Cognitive Assessment; IHDS, International HIV Dementia Scale.

In patients with MoCA scores < 26 and IHDS ≤ 10, 17 (54.8%) and 14 (63.6%) patients respectively, had previous opportunistic infections. In the cognitively-well group (MoCA ≥ 26 and IHDS > 10), only 4 patients had previous opportunistic infections.

Table 6 summarises the psychiatric co-morbidities of the sample. The most common co-morbidity was a substance use disorder (63.7%), with methamphetamine as the most commonly used illicit substance (53.3%) (Table 7). The diagnosis of HAND was made in only 12.7% of patients.

**TABLE 6:** Psychiatric diagnoses in women living with HIV (*n* = 55).

Psychiatric diagnosis	*n*	%
**Neurodevelopmental disorder**	3	5.5
**Mood disorders**		
Major depressive disorder	2	3.6
Bipolar 1 disorder	12	21.8
Bipolar disorder because of HIV	3	5.5
**Psychotic disorders**		
Schizophreniform disorder	2	3.6
Schizophrenia	6	10.9
Substance induced psychotic disorder	4	7.3
Psychotic disorder because of HIV	15	27.3
Schizoaffective disorder	11	20.0
**Substance use disorder**	35	63.7
**HAND**	7	12.7

HAND, HIV-associated neurocognitive disorder.

**TABLE 7:** Substance of choice for women living with HIV who reported using substances (*n* = 43).

Substance	*n*	%†
Cigarettes	37	86.0
Alcohol	16	37.2
Methamphetamine	23	53.5
Cannabis	21	48.8
Methaqualone	13	30.2

† , Percentages calculated to the total sample size (*n* = 43).

Only 3 patients (5.5%) refused ART and were discharged without an ART regimen. The majority of patients were on ART in the past (76.4%). An index diagnosis of HIV was made in 12 patients who were all initiated on ART during the admission. A total of 34 (61.8%) out of 55 patients were discharged on the fixed drug combination (one tablet daily); 15 (27.3%) patients on first–line ART, but not the fixed drug combination; and 3 (5.5%) patients on second-line ART.

All 55 patients were discharged on psychotropic medication and almost half were also on medication for side effects or medical comorbidities. Most patients (94.5%) were discharged on an antipsychotic and 50.9% of patients were discharged on a mood stabiliser. A total of 47 patients (74.5%) needed to take treatment twice daily. The mean number of tablets taken per day per patient was 7.71, with a minimum of 1 tablet daily and a maximum of 22 tablets daily. Side effects were experienced by 25.5% of patients, with extra pyramidal side effects being the most common (20%).

## Discussion

This 1-year retrospective review provides information on the socio-demographic and clinical characteristics of WLWH admitted to an acute psychiatric unit.

Of the 347 patients admitted and tested for HIV during the 1-year time period, only 55 (15.85%) patients tested positive for HIV, which is lower than the 25% prevalence rate reported for South African females aged 15 to 64 years.^1^ This finding was unexpected because the prevalence of HIV in psychiatric patients tends to be higher than in the general population.^5,6,7^ A recent study conducted at Lentegeur Hospital in the Western Cape reported that 28% of female acute psychiatric inpatients were living with HIV.^34^ One possible explanation for the difference between Stikland and Lentegeur could be that the area (Khayelitsha) that was reported to have the most HIV-related deaths in the Cape Metro region is covered by Lentegeur.^35^

The mean age of the cohort was 30.7 years, which is slightly higher than the median age of 27.6 years for women in South Africa.^36^ Most patients in our study were unmarried (78.2%) which is similar to studies by Collins et al. and Uys et al.^7,26^ Although few patients were married, most (83.6%) of the patients lived with a family member. This is encouraging for treatment strategies because a supportive family environment plays an important role in coping,^37^ adaptation to illness^38^ and utilisation of health services. In this cohort, 61.8% of the participants had children. Greater childcare burden is known to influence treatment outcomes in PLWH.^39^ Overall, it is recommended that clinicians should consider household composition and childcare burden and possibly provide educative support to families to improve HIV-treatment outcomes.

Most patients (58.2%) were educated to Grade 8–10 and only 10.9% had completed Grade 11 and 12. This correlates with a similar South African study which showed that 53% of patients had secondary school education up to Grade 11.^26^ The proportion of patients who were unemployed (92.7%) is much higher than the current South African unemployment rate of 29.1%.^40^ Franken et al. found a similar unemployment rate of 89% amongst female patients admitted to another acute psychiatric unit in the Western Cape.^34^

Only 3 of our 55 patients (5.5%) had a history of interpersonal violence documented in their folders, which is low given that it has been reported that one in three South African women experience physical interpersonal violence at some point in their current relationship.^41^ Most of the female patients in this study were involuntary patients under the *Mental Health Care Act* and may have felt too anxious or vulnerable to disclose their history. Reduced disclosure in the context of HIV is concerning because interpersonal violence can cause decreased willingness to access health services.^42^ Additionally, no data for history of interpersonal violence were documented in 14.5% of folders, a finding that highlights the need for systematic and sensitive screening for interpersonal violence in the local setting.

The average length of stay during the current admission was 65.6 days, which is much longer than reported elsewhere.^43^ This situation could be explained by the fact that the other study was conducted in a psychiatric setting in a general hospital, rather than in a psychiatric hospital. Because of limited numbers of step-up/step-down beds available, patients who are not ready to go home and function independently are often transferred to psychiatric hospitals where they are kept longer in acute inpatient settings, as the only option available. This calls to attention the great need for the provision of transitional interventions and facilities in South Africa.

Only 25 of the 55 patients had their viral load measured. Twelve patients were initiated on ART during their current admission and therefore their viral load was not measured as per current guidelines. Clinician error could also have contributed to limited viral load testing in those patients already on ART. The average most recent viral load for 25 patients was 30 703.8 copies/mL and the highest viral load was 36 7164 copies/mL. Our findings are concerning because high viral load is known to negatively affect HIV treatment outcomes, especially in terms of medication adherence.^18,44^ The small number of patients who were optimally virally suppressed (8 in total) might highlight the need to improve medication adherence in this population.

Higher viral loads often correlate with lower CD4 counts and more rapid disease progression, which includes the development of opportunistic infections.^45^ Opportunistic infections occurred in 23 patients (41.8%). The prevalence of syphilis (20%) in our cohort was much higher than the estimated prevalence of 0.5% in the general population.^46^ Franken et al. also found a relatively high prevalence of syphilis (12.5%) amongst female patients with acute mental illnesses.^34^ Patients with severe mental illness are known to engage in high-risk behaviours that put them at risk for contracting sexually transmitted diseases like syphilis.^8^ Syphilis and HIV are known to increase the acquisition and affect the clinical course of one another.^47^ Neurosyphilis itself can cause cognitive impairment and other psychiatric symptoms.^48^

The average most recent CD4 count of our 54 patients was 620.9 cells/mm^3^ which compares with the CD4 count of 674 cells/mm^3^ reported in a similar study.^43^ However, these statistics are much higher than those reported elsewhere. Where Uys et al. found that the average CD4 count was 186 cells/mm^3^ in patients with a psychiatric disorder because of HIV and 366 cells/mm^3^ in patients who had HIV and a primary psychiatric disorder.^26^ More patients in our study were previously on ART, as per the current guidelines, compared to those in the study by Uys et al. which could account for the difference between studies.^26^ Unexpectedly, figures for average CD4 count and average viral load were both high in our study, but this could likely be accounted for by a few very high viral load outliers.

Only 34 (61.8%) out of 55 participants were on the fixed drug combination (one tablet daily) despite this being a far less complicated ART regimen than others. A possible reason for this could be that the fixed drug combination initiated during and preceeding the study period included efavirenz, which had the potential for neuropsychiatric side effects. Clinicians were therefore often reluctant to start efavirenz in patients known with psychiatric disorders.^49^ In the context of HIV, individuals with cognitive impairment may have greater difficulty managing complex treatment regimens than those without cognitive impairment.^50^ It is therefore important to diagnose cognitive impairment in PLWH to ensure optimal treatment outcomes and to simplify treatment regimens if possible. Hopefully, the replacement of efavirenz with dolutegravir in the fixed drug combination^33^ will lead to more patients with psychiatric disorders initiated on this regimen.

The MoCA was completed for 37 of the 55 patients and the IHDS for 34 patients. The remaining patients may have been too psychotic, manic or depressed prior to discharge which would have precluded cognitive testing. We found a mean MoCA score of 18.1 (SD 5.6) and 32 patients scored lower than 26, indicating cognitive impairment. The mean IHDS score was 9.6 (SD 1.6) and 22 patients had scores ≤ 10. There is limited literature on MoCA and IHDS screening in PLWH admitted with mental health illness, so it was not possible to compare our results with other studies. Nevertheless, studies have shown that cognitive impairment within the domains of executive functioning, learning and memory, attention and global cognitive functioning are associated with poorer treatment outcomes, such as poor medication adherence.^51^

Although 32 patients scored lower than 26 on the MoCA, only 4 of these patients were diagnosed with HAND. Of the 22 patients who had IHDS scores ≤ 10, only 6 patients were diagnosed with HAND upon discharge. We acknowledge that a diagnosis of HAND cannot be made using only the MoCA or IHDS. Subjective or collateral complaints of cognitive impairment as well as objective evidence on cognitive testing should be included.^52^ In our study, there may not have been enough evidence of cognitive symptoms on history to make the diagnosis. Furthermore, even though the cognitive testing was done when patients were euthymic and apsychotic, the presence of residual mood or psychotic symptoms might have complicated the interpretation of these tests and made the clinician reluctant to diagnose a cognitive disorder. It is well known that HAND complicates the management of known psychiatric conditions in a variety of ways, including affecting adherence negatively,^50^ causing diagnostic challenges and causing drug–drug interactions, and increases the susceptibility to medication side effects.^53^ Therefore, the diagnosis of HAND is important for effective treatment planning in this population, and it is recommended that clinicians have more of an awareness to screen for HAND.

Although cognitive impairment in PLWH is known to be associated with higher viral loads^54^ and lower nadir CD4 counts,^55^ we found no significant association between these variables. This could be because of the small size of our sample, in which a few patients had a very high viral load compared to the rest of the cohort. More patients in the cognitively impaired group had previous opportunistic infections compared to the cognitively-well group. The presence of opportunistic infections could indicate previous World Health Organization Stage 4 disease and neuronal injury which can lead to cognitive impairment.^56,57^

The most common psychiatric diagnosis in our sample was a substance use disorder (63.7%), and substance abuse was reported by 78.2% of the study population. Methamphetamine was the most used illicit substance (53.5%). Other substances of abuse included nicotine (86%), cannabis (48.8%) and alcohol (37.2%). Our findings agree with the current knowledge that substance abuse is very common in the Western Cape with the aforementioned three being the most commonly used substances.^58^ Interestingly, our figures are much higher than those reported by Franken et al. in a similar setting, who showed that 38% of female acute psychiatric inpatients used substances, with cannabis (26%) and methamphetamine (22%).^34^ This could possibly be explained by the fact that our sample consisted only of patients with HIV, which in itself is concerning given that substance abuse is known to reduce ART adherence^59^ and may contribute to cognitive impairment.^60^

Other psychiatric disorders in our cohort were a psychotic disorder because of HIV (27.3%), bipolar I disorder (21.8%) and schizoaffective disorder (20%). Studies have shown an increased risk of medication non-adherence in PLWH with comorbid psychiatric disorders, such as depression, substance use disorders and anxiety disorders.^22^

A limitation of this retrospective study is that our data depended on how accurate and comprehensive the data were in the patient folders. A limitation is that the MoCA has not yet been validated or adapted in South Africa which means that our patients may have scored more poorly on screening than their actual cognitive ability. Furthermore, only 10.9% of patients in our study completed 12 years of education. Even though 1 point was added to the total MoCA score in patients with less than 12 years of education (as per the MoCA instructions), the level of education could have had an impact on the results. Viral load, CD4, MoCA and IHDS tests were not done on all participants which may limit the accuracy of our findings. The specific time point that the CD4 count, and the viral load tests were done was not specified and this could have influenced the interpretation of results. The number of PLWH and admitted to an inpatient psychiatric unit was relatively small. Lastly, the retrospective nature of our study limits the inference of causality. We recommend that future studies, with larger sample sizes, explore the link between certain demographic factors and treatment outcomes for WLWH and who are admitted with acute mental health illness.

## Conclusion

This study highlighted the socio-demographic and clinical characteristics of WLWH discharged from an acute psychiatric unit. Prominent demographic features included an average age of 30.7 years, being unmarried, being unemployed, living with a family member and being educated to a secondary level (Grade 8–10). Substance abuse was prevalent amongst patients, and the most common psychiatric diagnosis upon discharge was that of substance use disorder. The high percentage of patients initiated on ART (21.8%) during admission, the overall poor performance in cognitive screening and the minority of patients with viral suppression indicate the importance of developing group-specific management strategies. Our findings may inform service planning and emphasise the need for targeted intervention strategies that improve treatment outcomes in this vulnerable group.
